# Potential role for mucosally active vaccines against pneumococcal pneumonia

**DOI:** 10.1016/j.tim.2009.12.001

**Published:** 2010-02

**Authors:** Kondwani C. Jambo, Enoch Sepako, Robert S. Heyderman, Stephen B. Gordon

**Affiliations:** 1 Malawi-Liverpool-Wellcome Trust Clinical Research Programme, P.O. Box 30096, Chichiri, Blantyre, Malawi; 2 Respiratory Infection Group, Liverpool School of Tropical Medicine, Pembroke Place, Liverpool, L3 5QA, UK; 3 School of Clinical Sciences, University of Liverpool, Daulby Street, Liverpool, L69 3GA, UK

## Abstract

Pneumococcal pneumonia is a life-threatening disease with high mortality and morbidity among children under 5 years of age, the elderly and immunocompromised individuals worldwide. Protection against pneumococcal pneumonia relies on successful regulation of colonisation in the nasopharynx and a brisk alveolar macrophage-mediated immune response in the lung. Therefore, enhancing pulmonary mucosal immunity (which includes a combination of innate, humoral and cell-mediated immunity) through mucosal vaccination might be the key to prevention of pneumococcal infection. Current challenges include a lack of information in humans on mucosal immunity against pneumococci and a lack of suitable adjuvants for new vaccines. Data from mouse models, however, suggest that mucosally active vaccines will enhance mucosal and systemic immunity for protection against pneumococcal infection.

## Prevention of pneumococcal pneumonia: current strategies

**Figure 1:**
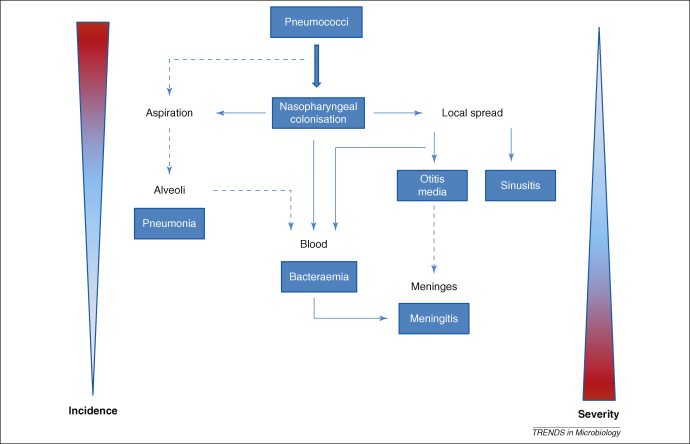
Diseases caused by *Streptococcus pneumoniae*. Pneumococci colonise the nasopharynx, evade host immunity and spread to the middle ear, sinus, lower respiratory tract, blood and meninges. Pneumococci cause otitis media in the middle ear, sinusitis in the sinus, pneumonia in the lower respiratory tract, bacteraemia in blood and meningitis in the meninges. The incidences of different types of pneumococcal infection are inversely related to the severity of disease: otitis media is the most common but the least severe. Redrawn and redesigned with permission from Ref. [2].

*Streptococcus pneumoniae* (the pneumococcus) is a Gram-positive aerobic commensal bacterium which forms part of the normal flora in the nasopharynx [1]. The pneumococcus can evade the immune system through a combination of surface expressed and secreted virulence factors to cause mucosal diseases such as otitis media, sinusitis and pneumonia, as well as systemic diseases such as bacteraemia and meningitis [1,2]. These diseases, collectively termed pneumococcal disease, can be classified as invasive or non-invasive disease. Otitis media, sinusitis and non-bacteraemic pneumococcal pneumonia are examples of non-invasive disease which are confined to the mucosal surface, whereas bacteraemic pneumonia, bacteraemia and meningitis are examples of invasive disease. Bacteraemic pneumococcal pneumonia, defined as having pneumonia and a positive blood culture [3], is more common in HIV-infected patients. Invasive pneumococcal disease is thought to progress from colonisation to bacteraemia, with or without pneumonia, only a minority of cases developing meningitis (Figure 1).

Pneumonia accounts for 19% of all under 5 year old deaths worldwide, which makes it the most deadly infectious illness for this age group [4]. The pneumococcus is the leading cause of pneumonia in children and it has been reported to cause over 50% of severe pneumonia cases in Africa [4]. Pneumococcal disease is most prevalent in the young and the elderly, but is also very common among HIV-infected individuals, who are 20–40 times more likely than uninfected adults to suffer from this illness [5].

Pneumococcal pneumonia is treatable using antibiotic therapy. However, where treatment is delayed or unavailable mortality is high [5]. Previously, the developing world had focused on *treating* pneumococcal disease rather than *preventing* it, but with the current increase in antibiotic resistance and the HIV pandemic, it is widely accepted that prevention is the key to minimising the disease burden [5].

Vaccination offers the most efficient and cost-effective method of preventing this disease. However, there are more than 90 pneumococcal serotypes which make development of a vaccine to provide universal protection a big challenge. There are two formulations of pneumococcal vaccines that have been licensed thus far: polysaccharide vaccines (PPVs) and protein conjugate vaccines (PCVs). The 23-valent pneumococcal polysaccharide vaccine, which contains purified capsular polysaccharide antigens from 23 serotypes, offers some protection against invasive pneumococcal disease in adults but is not effective in either children less than 2 years of age or immunocompromised adults [6]. PCVs, which contain purified capsular polysaccharides conjugated to a carrier protein, offer protection against both pneumonia and invasive disease in children [7] and immunocompromised adults (French *et al.* unpublished). The currently licensed 7-valent conjugate vaccine (containing 7 capsular polysaccharides conjugated to a diphtheria CRM197 protein) is being used as part of childhood immunisation programmes in several countries but others are waiting for the licensing of 10-valent and 13-valent vaccines. The disadvantages of PCVs are that they are expensive, have limited serotype coverage, can be associated with an increase in disease caused by serotypes not included in the vaccine and are less effective against radiological pneumonia (20–37% efficacy) [7,8] than against invasive disease (77–83% efficacy) [7]. In African children, PCVs appear to provide no protection to unvaccinated children (herd immunity) and is not very effective against colonisation (39% against vaccine serotypes, 0% against all serotypes) [9].

There are several key developments that would result in a breakthrough in the global control of pneumococcal disease. Use of the PCV is an important landmark [8] but the use of conserved proteins in a universal vaccine would allow a single vaccine to be deployed in all geographical regions without regard to the prevalent serotype patterns. In addition, the development of mucosally active vaccines might reduce mucosal disease including pneumonia and otitis media, but this will require the identification of safe and effective mucosal adjuvants for successful vaccine delivery.

This review will focus on recent advances in our understanding of mucosal immunity relevant to pneumococcal infection and, in particular, the critical immune responses that must be augmented by new vaccines.

## Mucosal immunity against *Streptococcus pneumonia*

### Host response against pneumococcal colonisation

Pneumococcal colonisation of the upper respiratory tract precedes infection of the lower respiratory tract, but is normally asymptomatic and not usually followed by disease [2]. The local host immune response plays an important role in regulating the containment of pathogens within the nasopharyngeal cavity [2]. A brisk local host immune response to *S. pneumoniae* involving phagocytes (neutrophils and macrophages), B cells (antibodies against pneumococcal polysaccharides and proteins) and T cells rapidly eliminates colonisation, whereas a poor mucosal immune response results in protracted colonisation [2]. Both innate and adaptive immunity play a role in these host defence responses against *S. pneumoniae*.

### Innate immune response during colonisation

Innate factors (including C-reactive protein, CRP) play a crucial role in the host defence against colonisation with *S. pneumoniae*. CRP is an acute-phase protein which is mainly found in serum and elevated during inflammation [10]. It has also been detected in the upper respiratory tract of healthy individuals and is also elevated during inflammation [11]. The concentration of CRP is lower in the respiratory tract than in serum but is sufficient to contribute to innate immunity, and is locally produced by epithelial cells [11,12]. CRP has several functions in relation to cell-surface phosphorylcholine-expressing bacteria (such as *S. pneumoniae* and *Haemophilus influenzae*), which includes activation of complement by the classical pathway, enhancing opsonisation and inhibition of the attachment of bacteria to epithelial cells [12]. These functions help in clearance of colonising pneumococci from the upper respiratory tract, which if not cleared might transmigrate across endothelial and epithelial monolayers in a process called pericellular tissue invasion [13].

It is known that complement plays a role in protection against pneumococcal infection through the promotion of opsonophagocytosis [14,15]. However, there are still conflicting data on the role of complement in protection against colonisation. Data from a co-colonisation mice model (of *S. pneumoniae* and *H. influenzae*) suggest that successful clearance of pneumococci in the nasopharynx resulted from opsonisation by complement, followed by phagocytosis by neutrophils which were recruited to the mucosal surface [16]. In a different model, C3 (a protein that plays a central role in activation of the complement system) was essential within the lungs for an optimal immune response against *S. pneumoniae* and subsequently played an important role systemically [17]. In contrast, another murine colonisation model showed that C3-deficient mice lacking functional complement system cleared pneumococcal colonisation at the same rate as wild type mice [18]. It is important to take note that murine models vary according to the genetic background of the mouse and the serotype of pneumococcus used, and hence they might not always produce similar results.

The innate response includes cellular responses from neutrophils and macrophages. It has been demonstrated in murine models that pneumococcal colonisation of the upper respiratory tract triggers an acute inflammatory response characterised by a robust influx of neutrophils into the lumen of the paranasal spaces [18,19] and release of cytokines (tumour necrosis factor alpha, TNF-α) and chemokines (interleukin 8, IL-8) [20]. This acute inflammatory response is ineffective at controlling initial mucosal colonisation [19], but it enhances the adaptive immune response and subsequent bacterial clearance [21].

### Adaptive immune response during colonisation

Nasopharyngeal colonisation stimulates the production of secretory IgA antibodies (the predominant immunoglobulin class in human external secretions) and serum IgG [22]. However, it is unclear whether these antibodies are protective against colonisation. Many isolates of *S. pneumoniae* secrete a zinc metalloprotease which inactivates IgA1 (a subclass of IgA). Furthermore, the cleaved IgA1 fragment might assist translocation of the opsonised bacteria across the host respiratory epithelium [23]. By contrast, increased concentrations of serotype-specific antibodies against pneumococcal polysaccharides [24,25] and antibodies against pneumococcal proteins in serum and saliva [26] have been correlated with increased protection against carriage. These antibodies opsonise pneumococci, making it easier for phagocytes to recognise, ingest and clear bacteria from the respiratory tract [27]. This has long been thought to be the primary main mechanism for protection against pneumococcal colonisation.

**Figure 2:**
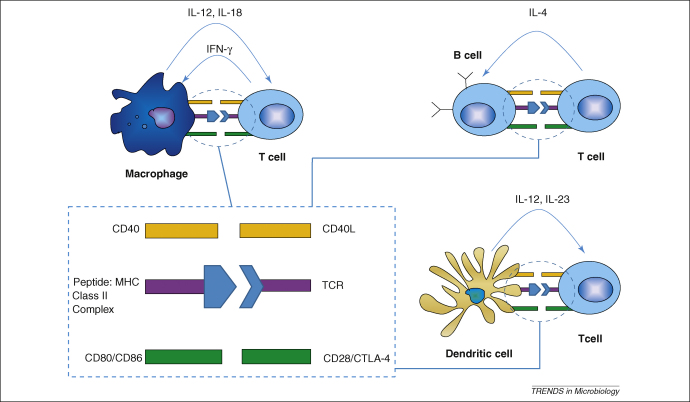
Induction of cell-mediated immune response by CD4^+^ T cells. There are three signals that are important during T cell activation: antigen presentation (TCR:Peptide-MHC class II ligation), co-stimulation (CD40:CD40L and CD80/CD86:CD28) and polarising signals (cytokine milieu). Professional antigen-presenting cells (dendritic cells, B cells and macrophages) present antigens to T cells in the context of MHC class II via the TCR (T cell receptor). This induces upregulation of CD40L and CD28 on the T cells, which bind to their receptors CD40 and CD80/CD86, respectively, on APCs, in a process called co-stimulation. These events lead to the production of polarising cytokines by APCs which include IL-12 from macrophages and dendritic cells, and IL-23 from dendritic cells. The polarising cytokines are important because they dictate the fate of T cells on whether to differentiate into Th1, Th2 or Th17.

Several recent studies suggest that other mechanisms of protection against pneumococcal carriage are required, in addition to antibody-mediated immunity. Firstly, the course of an experimental colonisation is not affected in mice that are unable to produce pneumococcal-specific antibody [28]. Secondly, the adaptive immune response is enhanced in the presence of pneumolysin and neutrophils. Pneumolysin, a pore-forming cytotoxin is a critical pathogenic factor of *S. pneumoniae* (Box 1). The interaction of pneumolysin and neutrophils promotes delivery and release of pneumococcal-specific antigens to the nasal associated lymphoid tissues, a process that is impaired in either neutrophil- or pneumolysin-deficient conditions [21]. Impaired antigen delivery is associated with prolonged nasopharyngeal colonisation [21]. Finally, mice lacking the ability to induce a cell-mediated immunity owing to the absence of appropriate molecules to present antigens to CD4^+^ T cells (MHC-II knockout mice) show prolonged carriage, suggesting an important role for CD4^+^ T cells rather than antibody-mediated immunity [29] (for the antigen presentation process, see Figure 2).

Studies have shown that immunity to pneumococcal colonisation is mediated by a specific subset of CD4^+^ T cells (Th17) which produce IL-17A [30–34]. Malley *et al.* showed that blocking IL-17A in mice models reduced immunity to pneumococcal colonisation following intranasal immunisation with cell wall polysaccharide [33]. In another study by the same group, they showed that IL-17A expression in peripheral blood samples from immunised mice was associated with protection *in vivo* against pneumococcal carriage [32].

IL-17A-mediated protection against pneumococcal colonisation results in recruitment of neutrophils into the upper airway lumen to clear bacterium [32,34]. Recently, Zhang *et al.* showed that primary challenge with pneumococci in mice generates CD4^+^ T cell memory, resulting in enhanced Th17-mediated recruitment of neutrophils after secondary pneumococcal challenge [34]. These neutrophils were shown to contribute to early bacterial clearance following secondary challenge [34]. However, it is still not clear whether Th17 cells are involved in immunity against human pneumococcal colonisation.

## Host response against pneumococcal pneumonia

### Post colonisation events leading to pneumonia

If pneumococci colonising the human nasopharynx are aspirated into the distal airways and alveolar air spaces, they will interact with pulmonary defence mechanisms. The bacteria will either be cleared or cause disease. Excessive replication of the bacteria in the alveoli triggers infiltration of immune cells which – if not properly regulated – impairs gas exchange resulting in the clinical syndrome of pneumonia.

The process of bacterial clearance in the lung is a highly regulated process – an excessive response might potentially lead to tissue damage, whereas a weak response leads to exponential growth of the pathogens. The primary host immune defence against small numbers of pneumococci during early infection is phagocytosis [27] which is enhanced through opsonisation by immunoglobulin and complement [19], in a process called opsonophagocytosis. The mechanism of host defence against pneumococci during late infection is different. It involves multiple immune cells and combination of innate and adaptive immunity.

### Early infection in the lung

Alveolar macrophages are the first cells that combat pneumococci during early infection [35] and the main cell population that mediates mucosal responses in the lower airways [36,37]. Approximately 90% of cells found in bronchoalveolar lavage fluid of healthy volunteers are macrophages [37]. It has been suggested that during early infection (where the bacterial load is low), resident alveolar macrophages are capable of multiple episodes of phagocytosis and killing [38]. This helps to clear the bacteria without recruitment of inflammatory cells such as neutrophils, and hence maintaining a low inflammatory state in the lung [38]. Early clearance of pneumococci through alveolar macrophage phagocytosis probably prevents bacteria–dendritic cell interaction, which in turn limits the initiation of T cell-mediated inflammatory responses in the lymph node [37].

It is still unclear whether antigen presentation occurs in the lung, in the draining lymph nodes, or both, and whether alveolar macrophages are part of this antigen presentation process. There is data *in vitro* to suggest that alveolar macrophages are able to present antigens to T cells, although less effectively than other antigen-presenting cells (APCs) [39]. Alveolar macrophages might induce antigen-specific unresponsiveness in CD4^+^ T cells as a result of antigen recognition in the absence of co-stimulation [39] (for the antigen presentation process, see Figure 2).

### Late infection in the lung

When the alveolar bacterial load rises above a critical threshold, alveolar macrophages cease to perform effective opsonophagocytosis and produce an increased proinflammatory cytokine response dominated by TNF-α and IL-8 [38]. The presence of TNF-α is not a prerequisite for pulmonary anti-pneumococcal responses, because successful clearance of the bacteria can occur independent of this cytokine – but it is beneficial during systemic infection [40]. Inflamed epithelial cells enhance neutrophil recruitment into the lung by secretion of IL-8 [41], both as a direct result of pneumococcal binding to epithelial receptors and in response to macrophage proinflammatory signalling.

When a proinflammatory signal (TNF-α and/or IL-8) is produced in the alveolus, there is upregulation of adherence molecules on endothelial cells which bind to their receptors on neutrophils. This results in rolling of neutrophils along the endothelial wall and transmigration into the alveolar space in a process called chemotaxis [42]. Neutrophils then become the major immune cell population responsible for pneumococcal clearance in the lung [43].

**Figure 3:**
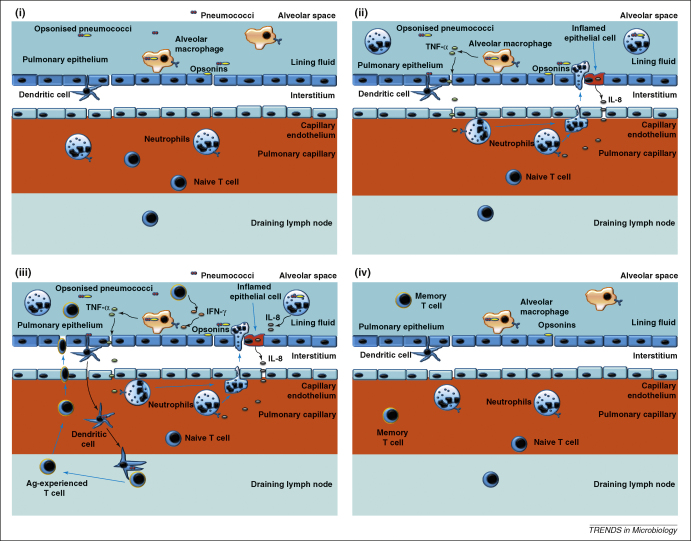
Pneumococcal clearance in the lung. Host defence in the lower respiratory tract is mediated by alveolar macrophages. **(i)** During early infection where the bacterial load is low, resident alveolar macrophages efficiently kill and phagocytise opsonised pneumococci in a quiescent manner, effectively preventing bacteria–dendritic cell interaction, and hence inhibiting initiation of T cell-mediated inflammatory responses. **(ii)** In situations where bacterial load exceeds the capability for macrophages to perform effective opsonophagocytosis, neutrophils are recruited following secretion of TNF-α by alveolar macrophages and/or IL-8 by epithelial cells. **(iii)** T cells are recruited following successful antigen presentation in the draining lymph nodes by pulmonary dendritic cells. These cells secrete IFN-γ which activates macrophages to kill internalised pneumococci and also promotes further TNF-α production by alveolar macrophages. **(iv)** Following clearance of pneumococci from the lungs, neutrophils, some macrophages and T cells undergo rapid apoptosis. Surviving T cells remain in the alveoli as resident effector memory cells.

T cells are also recruited in high numbers to the lung in late infection – the peak of T cell infiltration in the lung during intranasal pneumococcal infection in mice *in vivo* coincided with the phase when bacterial growth ceased [44]. The recruited T cells are predominantly of the effector memory phenotype and potentially secrete interferon gamma IFN-γ to activate alveolar macrophages. The actual mechanisms on the role played by T cells are still not clear, but we have hypothesised some of the possible pathways in Figure 3. In addition, it has been shown that T cells expressing the gammadelta receptor (γδ T cells) act as regulators of alveolar macrophages and pulmonary dendritic cells during the resolution of pneumococcus-mediated lung inflammation [45]. Cytoxicity mediated by γδ T cells helps restore mononuclear phagocyte numbers to homeostatic levels, and hence preventing excessive inflammation in the lung [45].

Following clearance of pneumococci from the lungs, neutrophils, some macrophages and T cells undergo rapid apoptosis. Macrophage apoptosis leads to reduced TNF-α expression, which in turn results in reduced neutrophil recruitment and enhanced neutrophil apoptosis [46]. Dead cells are then cleared through phagocytosis, efferocytosis (clearance of apoptotic cells by phagocytes) and the normal function of the mucociliary escalator, whereas the surviving T cells remain in the alveoli as resident effector memory cells.

In summary, mucosal responses are critical in regulating pneumococcal carriage and defence against infection. Therefore, enhancing pulmonary mucosal immunity might be an effective strategy in the prevention of pneumococcal disease.

## Evidence for vaccine-induced mucosal immunity against pneumococcus

Mucosal exposure to *S. pneumoniae* induces both mucosal and systemic humoral and cellular immune responses [25,26]. Enhancing these responses by mucosal vaccination is an attractive immunisation approach against pneumococcus as it mimics the natural route of infection. The success of other mucosal vaccines such as the trivalent oral poliovirus vaccine shows that this approach is a viable alternative of delivering vaccines [47]. There is evidence from mouse models, employing a similar route of vaccine delivery, showing that oral immunisation of PspA family fusion proteins delivered by attenuated *Salmonella enterica* serovar Typhimurium enhances protection against *S. pneumoniae* [48–51]. An important difference is that polio virus can replicate in the respiratory mucosa but pneumococcal antigens cannot. Nevertheless, there are several studies which have shown that mucosal immunisation can elicit protection against pneumococcal colonisation and infection (Table 1).

### Protection against pneumococcal colonisation

Mucosal immunisation of experimental animals has been shown to elicit protection against carriage [29,52]. Intranasal immunisation of mice with killed, unencapsulated, whole cell pneumococci and cholera toxin adjuvant elicited protection against experimental colonisation. The protection was dependent on CD4^+^ T cells and independent of antibody and bacterial serotype [29]. Furthermore, intranasal immunisation with a cholera toxin B subunit fused to the pneumococcal surface adhesin A (PsaA) also protected mice against colonisation with *S*. *pneumoniae* [52].

An ideal mucosal vaccine would include several pneumococcal proteins such as pneumolysin, PspA, PsaA or PspC [53]. Many of these proteins play a role in *S. pneumoniae* pathogenesis, and several are particularly relevant to protection against carriage [54]. Recently, Lu *et al.* demonstrated that a fusion conjugate, including cell wall polysaccharide coupled to pneumolysin and PsaA, delivered intranasally with cholera toxin, protected mice against experimental pneumococcal colonisation [31]. Mucosal vaccines based on protein combinations are more likely to exhibit coverage of all pneumococcal serotypes.

### Protection against pneumococcal pneumonia

Pneumococcal conjugate vaccine is less effective against pneumonia than against invasive pneumococcal disease [7,8]. This observation might be as a result of difficulty either in the diagnosis of non-bacteraemic pneumococcal pneumonia or in distinguishing this diagnosis from other infective causes of pneumonia. Consequently, the chances of underreporting non-bacteraemic pneumococcal pneumonia as an endpoint in determining efficacy of the pneumococcal conjugate vaccine are high. By contrast, the intramuscular administration of the vaccine might result in a compartmentalisation of the immune response and a suboptimal lymphocyte trafficking to the pulmonary mucosa.

Mucosal vaccination has shown promising results in protection against pneumococcal lung infection [31,55–57]. Nasal administration of *Lactococcus lactis* increased the clearance rate of *S. pneumoniae* from the lung and prevented invasion of pneumococci into blood [57]. In these experiments, *L. lactis* increased phagocyte activation in lung, blood and bone marrow of the vaccinated mice [57]. Moreover, mucosal immunisation with caseinolytic protease, a conserved pneumococcal protein, induced the production of both systemic and mucosal antibodies and resulted in reduced lung bacterial load in a pneumococcal pneumonia model and prevented death in an intraperitoneal sepsis model [58]. Lastly, a mucosal vaccine made of recombinant PspA elicited protection against invasive pneumococcal challenge, characterised by increased secretion of IL-17 and IFN-γ by lung and spleen cells, respectively [55]. These data support the concept that mucosal immunisation might protect against both mucosal and systemic infection. However, the duration of protection afforded by these mucosal vaccines is not clear.

There are also some data to suggest that mucosally administered vaccines might actually provide better protection against both mucosal and systemic disease than conventional parenteral (systemic) vaccines. Immunisation of mice with lactococcal PspA vaccine elicited better protection against respiratory pneumococcal challenge than conventional parenteral PspA vaccine in intraperitoneal sepsis and intranasal respiratory infection models [56].

In summary, there are encouraging data to support the role of mucosal vaccination in protection against both mucosal and systemic pneumococcal disease. However, lack of a suitable adjuvant is a major obstacle to success, but cytokine adjuvants might be useful [59].

## Concluding remarks and future directions

We have discussed the role of mucosal immunity and reviewed available data on mucosal immunisation against pneumococcal disease. There is evidence from murine studies to suggest that mucosal immunisation against pneumococci induces mucosal and systemic immunity more effectively than parenteral vaccination. The data from humans, however, are insufficient to draw firm conclusions. Further studies using lung, nasal and other mucosal samples from humans are needed.

Immediate priorities include the need to address the role of T cell-mediated immunity against pneumococcal colonisation and infection in humans. Such data might clarify the human correlates of protection or immunity to *S. pneumoniae*. These correlates of protection might then help in predicting efficacy to future vaccines. Research questions for future work in the field are outlined in Box 2.

Strategic decisions regarding future pneumococcal vaccines need to determine whether to focus on improving the current conjugate vaccines (by including more serotypes or replacing the carrier with a pneumococcal protein) or developing new pneumococcal protein based vaccines. Pneumococcal conjugate vaccines are currently being used in several developed countries because they provide good protection against systemic disease. Their main disadvantage is that they have limited serotype coverage and are less effective against mucosal disease. By contrast, pneumococcal protein based vaccines have the potential to offer universal coverage as well as offer protection against both mucosal and systemic disease if delivered through the mucosal route. Vaccination still remains the key to minimising the high burden of pneumococcal disease worldwide. We believe that alternative routes of immunisation (with conjugate vaccines or pneumococcal proteins) might help in improving the efficacy of pneumococcal vaccines to both mucosal and systemic disease.

## Figures and Tables

**Table 1:** Mouse models of mucosal immunisation with pneumococcal protein antigens^a^

Antigen or vaccine	Route	Immunogenicity	Correlates of protection	Protection against:	Refs
PspA	Intranasal	Mucosal and systemic	Antibodies in serum and saliva	Colonisation, pneumonia, sepsis	[60–62]
PspA/attenuated *Salmonella*	Oral	Mucosal and systemic	Antibodies in serum and vaginal fluids	Pneumonia, bacteraemia	[48–51,63]
PsaA	Oral	Mucosal and systemic	Antibodies in serum, BAL and intestinal fluid	Pneumonia, bacteraemia	[52,64,65]
PsaA/lactic acid bacteria	Intranasal	Mucosal and systemic	Antibodies in serum, saliva, nasal and bronchial washes	Colonisation	[66]
PotD	Intranasal	Mucosal and systemic	Antibodies in serum and saliva	Colonisation, pneumonia, bacteraemia	[67]
PsaA and PspA	Intranasal	Mucosal and systemic	Antibodies in serum and saliva	Colonisation	[68]
PspA and PspC	Intranasal	Mucosal and systemic	Antibodies in serum, vaginal washes, and BAL; cytokine responses in BAL, lung and splenic samples	Pneumonia, bacteraemia	[69,70]
PsaA, PdT and CWPS	Intranasal	Systemic	Antibodies in serum; T cell response in whole blood	Colonisation, pneumonia	[31,71]
GEM with PpmA, SlrA and IgA1p	Intranasal	Systemic	Antibodies in serum	Pneumonia	[72,73]
PCV	Intranasal	Mucosal and systemic	Antibodies in serum and nasal washes	Colonisation, otitis media	[74–77]

a Abbreviations: PspA, pneumococcal surface protein A; PsaA, ‘pneumococcal surface adhesion A’ protein; BAL, bronchoalveolar lavage; PotD, polyamine transport protein D; PspC, pneumococcal surface protein C; PdT, pneumolysin nontoxic derivative; CWPS, cell wall polysaccharide; GEM, Gram-positive enhancer matrix; PCV, pneumococcal conjugate vaccine.

## Box 1 The role of pneumolysin in pneumonia and bacteraemia

Pneumolysin is a potent pore-forming cytotoxin produced by almost all pneumococcal isolates, and a proven virulence factor. Animal studies show that this protein is required for the development of pneumonia and bacteraemia. Data suggests that *S. pneumoniae* requires pneumolysin to successfully survive in both the upper and lower respiratory tracts [1,78,79]. The cytotoxin is essential for pneumococcus to translocate from the lungs to the bloodstream [1,78–80]. It is also required for bacterial survival in blood: pneumolysin-expressing pneumococci are associated with high bacteraemia and severe infection [79,80]. By contrast, pneumolysin-deficient pneumococci result in low bacteraemia (often chronic) with minimal severity in animal models [81].

## Box 2 Questions for future research

• What are the correlates of protection (measurable signs of immunity) against carriage and disease as a result of *S. pneumoniae* in human mucosa?
• Which is the most effective mode of vaccine delivery to enhance both mucosal and systemic immunity to *S. pneumoniae*?
• What adjuvants are most effective for mucosal pneumococcal vaccine delivery?
• How does HIV infection affect mucosal immunity against *S. pneumoniae* in the respiratory tract?

## Glossary

Colonisation: establishment of bacteria in the upper respiratory tract without causing overt disease.
Compartmentalisation: in the context of this article, the regional sequestration of a specific function within the immune system. Local immune responses within the body (site of infection) are often functionally independent from systemic immunity.
Complement system: a cascade of plasma proteins that act together in defence against extracellular bacteria by coating bacteria (opsonisation) or killing pathogens directly.
Mucosal immunity: part of the immune system that is responsible for protection against pathogens, commensals and other non-microbial antigens restricted to the mucous membranes of the gastrointestinal, respiratory and urinogenital tracts, i.e. the major sites of antigen/pathogen entry into the body.
Opsonisation: alteration of surfaces of pathogens by immune molecules in such a manner that the pathogens are more easily recognised by immune cells.
Opsonophagocytosis: the process whereby bacteria are altered for optimal interaction with phagocytes and then engulfed.
Systemic immunity: the general immune system responsible for protection against pathogens, commensals and other non-microbial antigens; it is not confined to the site of infection or antigen entry.

## References

[1] Kadioglu A. (2008). The role of *Streptococcus**pneumoniae* virulence factors in host respiratory colonization and disease. Nat. Rev. Microbiol..

[2] Bogaert D. (2004). *Streptococcus pneumoniae* colonisation: the key to pneumococcal disease. Lancet Infect. Dis..

[3] Jover F. (2008). A comparative study of bacteremic and non-bacteremic pneumococcal pneumonia. Eur. J. Intern. Med..

[4] Wardlaw T. (2006). Pneumonia: The Forgotten Killer of Children.

[5] Turner D. (2008). Improving Global Health by Preventing Pneumococcal Disease.

[6] King J.C. (1996). Comparison of the safety and immunogenicity of a pneumococcal conjugate with a licensed polysaccharide vaccine in human immunodeficiency virus and non-human immunodeficiency virus-infected children. Pediatr. Infect. Dis. J..

[7] Cutts F.T. (2005). Efficacy of nine-valent pneumococcal conjugate vaccine against pneumonia and invasive pneumococcal disease in The Gambia: randomised, double-blind, placebo-controlled trial. Lancet.

[8] Qazi S. (2007). Global Action Plan for the Prevention and Control of Pneumonia (GAPP): Report of an Informal Consultation.

[9] Cheung Y.B. (2009). Nasopharyngeal carriage of *Streptococcus pneumoniae* in Gambian children who participated in a 9-valent pneumococcal conjugate vaccine trial and in their younger siblings. Pediatr. Infect. Dis. J..

[10] Du Clos T.W., Mold C. (2004). C-reactive protein: an activator of innate immunity and a modulator of adaptive immunity. Immunol. Res..

[11] Gould J.M., Weiser J.N. (2001). Expression of C-reactive protein in the human respiratory tract. Infect. Immun..

[12] Gould J.M., Weiser J.N. (2002). The inhibitory effect of C-reactive protein on bacterial phosphorylcholine platelet-activating factor receptor-mediated adherence is blocked by surfactant. J. Infect. Dis..

[13] Attali C. (2008). The interaction of *Streptococcus pneumoniae* with plasmin mediates transmigration across endothelial and epithelial monolayers by intercellular junction cleavage. Infect. Immun..

[14] Roy S. (2002). MBL genotype and risk of invasive pneumococcal disease: a case-control study. Lancet.

[15] Brown J.S. (2002). The classical pathway is the dominant complement pathway required for innate immunity to *Streptococcus pneumoniae* infection in mice. Proc. Natl. Acad. Sci. U. S. A..

[16] Lysenko E.S. (2005). The role of innate immune responses in the outcome of interspecies competition for colonization of mucosal surfaces. PLoS Pathog..

[17] Kerr A.R. (2005). Innate immune defense against pneumococcal pneumonia requires pulmonary complement component C3. Infect. Immun..

[18] van Rossum A.M. (2005). Host and bacterial factors contributing to the clearance of colonization by *Streptococcus pneumoniae* in a murine model. Infect. Immun..

[19] Nelson A.L. (2007). Capsule enhances pneumococcal colonization by limiting mucus-mediated clearance. Infect. Immun..

[20] Martner A. (2009). *Streptococcus pneumoniae* autolysis prevents phagocytosis and production of phagocyte-activating cytokines. Infect. Immun..

[21] Matthias K.A. (2008). Neutrophil-toxin interactions promote antigen delivery and mucosal clearance of *Streptococcus pneumoniae*. J. Immunol..

[22] O’Brien K.D. (2008). Nasopharyngeal Carriage.

[23] Weiser J.N. (2003). Antibody-enhanced pneumococcal adherence requires IgA1 protease. Proc. Natl. Acad. Sci. U. S. A..

[24] Weinberger D.M. (2008). Epidemiologic evidence for serotype-specific acquired immunity to pneumococcal carriage. J. Infect. Dis..

[25] Goldblatt D. (2005). Antibody responses to nasopharyngeal carriage of *Streptococcus pneumoniae* in adults: a longitudinal household study. J. Infect. Dis..

[26] Zhang Q. (2006). Serum and mucosal antibody responses to pneumococcal protein antigens in children: relationships with carriage status. Eur. J. Immunol..

[27] Gordon S.B. (2000). Intracellular trafficking and killing of *Streptococcus pneumoniae* by human alveolar macrophages are influenced by opsonins. Infect. Immun..

[28] McCool T.L., Weiser J.N. (2004). Limited role of antibody in clearance of *Streptococcus**pneumoniae* in a murine model of colonization. Infect. Immun..

[29] Malley R. (2005). CD4^+^ T cells mediate antibody-independent acquired immunity to pneumococcal colonization. Proc. Natl. Acad. Sci. U. S. A..

[30] Bogaert D. (2009). Impaired innate and adaptive immunity to *Streptococcus pneumoniae* and its effect on colonization in an infant mouse model. Infect. Immun..

[31] Lu Y.J. (2009). Protection against pneumococcal colonization and fatal pneumonia by a trivalent conjugate of a fusion protein with the cell wall polysaccharide. Infect. Immun..

[32] Lu Y.J. (2008). Interleukin-17A mediates acquired immunity to pneumococcal colonization. PLoS Pathog..

[33] Malley R. (2006). Antibody-independent, interleukin-17A-mediated, cross-serotype immunity to pneumococci in mice immunized intranasally with the cell wall polysaccharide. Infect. Immun..

[34] Zhang Z. (2009). Cellular effectors mediating Th17-dependent clearance of pneumococcal colonization in mice. J. Clin. Invest..

[35] Gordon S.B., Read R.C. (2002). Macrophage defences against respiratory tract infections. Br. Med. Bull..

[36] Stockley R., Brewis R.C. (1995). Humoral and cellular mechanisms. Respiratory Medicine.

[37] Riches, D.W.H. and Fenton, M.J. (2009) Monocytes, macrophages and dendritic cells of the lung. In *Murray and Nadel's Textbook of Respiratory Medicine* (Online version) (Mason, R.B., *et al.*, eds), Elsevier

[38] Marriott H.M., Dockrell D.H. (2007). The role of the macrophage in lung disease mediated by bacteria. Exp. Lung Res..

[39] Blumenthal R.L. (2001). Human alveolar macrophages induce functional inactivation in antigen-specific CD4 T cells. J. Allergy Clin. Immunol..

[40] Kirby A.C. (2005). The role played by tumor necrosis factor during localized and systemic infection with *Streptococcus pneumoniae*. J. Infect. Dis..

[41] Murdoch C. (1999). Functional expression of chemokine receptor CXCR4 on human epithelial cells. Immunology.

[42] Zarbock A., Ley K. (2008). Mechanisms and consequences of neutrophil interaction with the endothelium. Am. J. Pathol..

[43] Hirst R.A. (2004). The role of pneumolysin in pneumococcal pneumonia and meningitis. Clin. Exp. Immunol..

[44] Kadioglu A. (2000). Host cellular immune response to pneumococcal lung infection in mice. Infect. Immun..

[45] Kirby A.C. (2007). Pulmonary dendritic cells and alveolar macrophages are regulated by gammadelta T cells during the resolution of *S. pneumoniae*-induced inflammation. J. Pathol..

[46] Marriott H.M. (2006). Decreased alveolar macrophage apoptosis is associated with increased pulmonary inflammation in a murine model of pneumococcal pneumonia. J. Immunol..

[47] Onorato I.M. (1991). Mucosal immunity induced by enhance-potency inactivated and oral polio vaccines. J. Infect. Dis..

[48] Kang H.Y. (2002). Immune responses to recombinant pneumococcal PspA antigen delivered by live attenuated *Salmonella enterica* serovar Typhimurium vaccine. Infect. Immun..

[49] Li Y. (2009). Evaluation of new generation *Salmonella enterica* serovar Typhimurium vaccines with regulated delayed attenuation to induce immune responses against PspA. Proc. Natl. Acad. Sci. U. S. A..

[50] Nayak A.R. (1998). A live recombinant avirulent oral *Salmonella* vaccine expressing pneumococcal surface protein A induces protective responses against *Streptococcus pneumoniae*. Infect. Immun..

[51] Xin W. (2009). PspA family fusion proteins delivered by attenuated *Salmonella enterica* serovar Typhimurium extend and enhance protection against *Streptococcus pneumoniae*. Infect. Immun..

[52] Pimenta F.C. (2006). Intranasal immunization with the cholera toxin B subunit-pneumococcal surface antigen A fusion protein induces protection against colonization with *Streptococcus pneumoniae* and has negligible impact on the nasopharyngeal and oral microbiota of mice. Infect. Immun..

[53] Ogunniyi A.D. (2007). Development of a vaccine against invasive pneumococcal disease based on combinations of virulence proteins of *Streptococcus pneumoniae*. Infect. Immun..

[54] Ogunniyi A.D. (2007). Contributions of pneumolysin, pneumococcal surface protein A (PspA), and PspC to pathogenicity of *Streptococcus pneumoniae* D39 in a mouse model. Infect. Immun..

[55] Ferreira D.M. (2009). Characterization of protective mucosal and systemic immune responses elicited by pneumococcal surface protein PspA and PspC nasal vaccines against a respiratory pneumococcal challenge in mice. Clin. Vaccine Immunol..

[56] Hanniffy S.B. (2007). Mucosal delivery of a pneumococcal vaccine using *Lactococcus lactis* affords protection against respiratory infection. J. Infect. Dis..

[57] Medina M. (2008). Nasal administration of *Lactococcus lactis* improves local and systemic immune responses against *Streptococcus pneumoniae*. Microbiol. Immunol..

[58] Cao J. (2008). Mucosal immunization with purified ClpP could elicit protective efficacy against pneumococcal pneumonia and sepsis in mice. Microbes Infect..

[59] Wright A.K. (2008). Prospects for use of interleukin-12 as a mucosal adjuvant for vaccination of humans to protect against respiratory pneumococcal infection. Vaccine.

[60] Wu H.Y. (1997). Intranasal immunization of mice with PspA (pneumococcal surface protein A) can prevent intranasal carriage, pulmonary infection, and sepsis with *Streptococcus pneumoniae*. J. Infect. Dis..

[61] Campos I.B. (2008). Nasal immunization of mice with *Lactobacillus casei* expressing the Pneumococcal Surface Protein A: induction of antibodies, complement deposition and partial protection against *Streptococcus pneumoniae* challenge. Microbes Infect..

[62] Yamamoto M. (1997). Oral immunization with PspA elicits protective humoral immunity against *Streptococcus pneumoniae* infection. Infect. Immun..

[63] Park S.M. (2008). MyD88 signaling is not essential for induction of antigen-specific B cell responses but is indispensable for protection against *Streptococcus pneumoniae* infection following oral vaccination with attenuated *Salmonella* expressing PspA antigen. J. Immunol..

[64] Seo J.Y. (2002). Cross-protective immunity of mice induced by oral immunization with pneumococcal surface adhesin a encapsulated in microspheres. Infect. Immun..

[65] Areas A.P. (2004). Expression and characterization of cholera toxin B-pneumococcal surface adhesin A fusion protein in *Escherichia coli*: ability of CTB-PsaA to induce humoral immune response in mice. Biochem. Biophys. Res. Commun..

[66] Oliveira M.L. (2006). Induction of systemic and mucosal immune response and decrease in *Streptococcus pneumoniae* colonization by nasal inoculation of mice with recombinant lactic acid bacteria expressing pneumococcal surface antigen A. Microbes Infect..

[67] Shah P. (2009). Mucosal immunization with polyamine transport protein D (PotD) protects mice against nasopharyngeal colonization with *Streptococcus pneumoniae*. Exp. Biol. Med. (Maywood).

[68] Briles D.E. (2000). Intranasal immunization of mice with a mixture of the pneumococcal proteins PsaA and PspA is highly protective against nasopharyngeal carriage of *Streptococcus pneumoniae*. Infect. Immun..

[69] Ferreira D.M. (2009). Characterization of protective mucosal and systemic immune responses elicited by Pneumococcal Surface Proteins PspA and PspC nasal vaccines against a respiratory pneumococcal challenge in mice. Clin. Vaccine Immunol..

[70] Xin W. (2008). Analysis of type II secretion of recombinant pneumococcal PspA and PspC in a *Salmonella enterica* serovar Typhimurium vaccine with regulated delayed antigen synthesis. Infect. Immun..

[71] Arevalo M.T. (2009). Mucosal vaccination with a multicomponent adenovirus-vectored vaccine protects against *Streptococcus pneumoniae* infection in the lung. FEMS Immunol. Med. Microbiol..

[72] Audouy S.A. (2006). Lactococcus lactis GEM particles displaying pneumococcal antigens induce local and systemic immune responses following intranasal immunization. Vaccine.

[73] Audouy S.A. (2007). Development of lactococcal GEM-based pneumococcal vaccines. Vaccine.

[74] Sabirov A., Metzger D.W. (2008). Intranasal vaccination of infant mice induces protective immunity in the absence of nasal-associated lymphoid tissue. Vaccine.

[75] Sabirov A., Metzger D.W. (2006). Intranasal vaccination of neonatal mice with polysaccharide conjugate vaccine for protection against pneumococcal otitis media. Vaccine.

[76] Lynch J.M. (2003). Increased protection against pneumococcal disease by mucosal administration of conjugate vaccine plus interleukin-12. Infect. Immun..

[77] Jakobsen H. (1999). Intranasal immunization with pneumococcal polysaccharide conjugate vaccines protects mice against invasive pneumococcal infections. Infect. Immun..

[78] Kadioglu A. (2002). Upper and lower respiratory tract infection by *Streptococcus pneumoniae* is affected by pneumolysin deficiency and differences in capsule type. Infect. Immun..

[79] Orihuela C.J. (2004). Tissue-specific contributions of pneumococcal virulence factors to pathogenesis. J. Infect. Dis..

[80] Berry A.M. (1999). Comparative virulence of *Streptococcus pneumoniae* strains with insertion-duplication, point, and deletion mutations in the pneumolysin gene. Infect. Immun..

[81] Benton K.A. (1995). A pneumolysin-negative mutant of *Streptococcus pneumoniae* causes chronic bacteremia rather than acute sepsis in mice. Infect. Immun..

